# Supplementary material for: Sex-Specific Differences in Adipose IRF5 Expression and Its Association with Inflammation and Insulin Resistance in Obesity
Source: Int J Mol Sci. 2025 Aug 25;26(17):8229. doi: 10.3390/ijms26178229 (PMC12428239; doi:10.3390/ijms26178229)
Supplement: Supplementary file 1 [file ijms-26-08229-s001.zip › ijms-3813220-supplementary.pdf]

Supplementary Table S1: Anthropometric, clinical, and biochemical characteristics of the study participants.

Physical and biochemical characteristics	Male (Mean ± SD)	Female (Mean ± SD)
Age (years)	44 ± 0.8	43 ± 2.39
Weight (kg)	96.50 ± 13.43	80.95 ± 11.68
Height (m)	1.74 ± 0.011	1.56 ± 0.06
BMI (kg/m2)	31.84 ± 0.90	33.10 ± 4.28
Waist circumference(cm)	107.7 ± 11.52	98.02 ± 13.27
Body Fat (%)	33.04 ± 1.04	41.05 ± 3.14
Triglycerides (mmol/l)	1.36 ± 0.88	1.26 ± 0.65
Total cholesterol (mmol/l)	4.88 ± 0.86	5.09 ± 1.06
LDL (mmol/l)	3.15 ± 0.69	3.24 ± 0.99
HDL cholesterol (mmol/l)	1.14 ± 0.25	1.29 ± 0.34
Fasting blood glucose (mmol/l)	5.33 ± 0.73	5.32 ± 0.72
HbA1c (%)	5.70 ± 0.53	5.61± 0.59

Supplementary Table S2. List of primers for qRT-PCR.

Gene	Assay ID	Gene	Assay ID	Gene	Assay ID
IL-1β	Hs01555410_m1	CCL8	Hs04187715_m1	CD141	Hs00264920_s1
IL-2	Hs00174114_m1	CCL-11	Hs00237013_m1	CD163	Hs00174705_m1
IL-5	Hs01548712_g1	CCL-15	Hs00361122_m1	CD302	Hs00994886_m1
IL-6	Hs00985639_m1	CCL18	Hs00268113_m1	TLR2	Hs01872448_s1
IL-8	Hs00174103_m1	CCL-19	Hs00171149_m1	TLR3	Hs01551078_m1
IL-10	Hs00961622_m1	CCL-20	Hs01011368_m1	TLR4	Hs00152939_m1
IL-12A	Hs01073447_m1	CXCL9	Hs00171065_m1	TLR7	Hs01933259_s1
IL-13	Hs00174379_m1	CXCL10	Hs01124251_g1	TLR8	Hs00152972_m1
IL-18	Hs01038788_m1	CXCL-11	Hs04187682_g1	TLR9	Hs00370913_s1
IL-33	Hs00369211_m1	IL-1RL1	Hs00545033_m1	TLR10	Hs01935337_s1
TNF-α	Hs01113624_g1	IL-2RA	Hs00907779_m1	IRF3	Hs01547283_m1
TGF-β	Hs00820148_g1	CCR1	Hs00928897_s1	IRF4	Hs01056533_m1
IFNB1	Hs01077958_s1	CCR2	Hs00704702_s1	IRF5	Hs00158114_m1
Dectin	Hs01902549_s1	CCR5	Hs99999149_s1	MyD88	Hs01573837_g1
SRA1	Hs00398296_g1	CD11c	Hs00174217_m1	IRAK1	Hs01018347_m1
CCL2	Hs00234140_m1	CD16	Hs04334165_m1	TRAF6	Hs00371512_g1
CCL3	Hs04194942_s1	CD68	Hs02836816_g1	NFKB	Hs00765730_m1
CCL5	Hs00982282_m1	CD86	Hs01567026_m1	GAPDH	Hs03929097_g1
CCL-7	Hs00171147_m1	RUNX	Hs02558380_s1	FOXP1	Hs00908900_m1
